# Screening for Fabry’s disease in a high-risk subpopulation of FMF

**DOI:** 10.1186/s40001-022-00846-1

**Published:** 2022-10-21

**Authors:** Tomer Maller, Ilan Ben-Zvi, Merav Lidar, Avi Livneh

**Affiliations:** 1 Medicine F, The Chaim Sheba Medical Center, Tel Hashomer, Ramat Gan, Israel; 2 FMF Clinic, The Chaim Sheba Medical Center, Tel Hashomer, Ramat Gan, Israel; 3 Rheumatology Unit, The Chaim Sheba Medical Center, Tel Hashomer, Ramat Gan, Israel; 4 The Sackler Faculty of Medicine, Tel Aviv University, Tel Aviv, Israel; 5 The Talpiot Medical Leadership Program, The Chaim Sheba Medical Center, Tel Hashomer, Ramat Gan, Israel

**Keywords:** Familial Mediterranean fever, Fabry’s disease, Comorbidities, Colchicine, Adverse effects, Misdiagnosis

## Abstract

**Background:**

Familial Mediterranean fever (FMF) is an autosomal recessive disease associated with mutations in the Mediterranean fever gene (MEFV) that manifests with recurrent episodes of febrile serositis. Fabry’s disease (FD) is an X-linked lysosomal storage disease caused by mutations in the *alpha-galactosidase A* gene and presents with a wide range of gastrointestinal, skin, vascular, renal and neurological manifestations. FMF and FD share similar manifestations, which may lead to misdiagnosis of one as the other; mostly FD is misdiagnosed as FMF. Moreover, various overlapping manifestations may stem from comorbidities, commonly coupled to FMF (such as Behcet's disease, inflammatory bowel disease, glomerulonephritis, fibromyalgia, and multiple sclerosis), as well as from colchicine adverse effects, which may add to the diagnostic confusion. Thus, we postulated that screening FMF for FD will lead to the identification of patients falsely diagnosed with FMF or who, in addition to FMF, suffer from FD that was previously missed.

**Methods:**

To identify missed FD among the FMF population, we performed chemical and genetic analyses for FD in blood samples obtained from a cohort of FMF patients followed in the specialized FMF center of our institution. To increase the likelihood of detecting patients with FD, we enriched the surveyed FMF population with patients exhibiting manifestations shared by patients with FD or who deviate from the typical FMF presentation.

**Results and conclusions:**

Of 172 surveyed FMF patients in a cohort derived from a clinic dedicated to FMF, none had FD. Thus, the postulation of increased odds for detecting FD in patients with FMF was not confirmed. Further exploration for FD in FMF population, is nevertheless recommended.

## Background

Familial Mediterranean fever (FMF) is an autosomal recessive autoinflammatory disease that manifests with attacks of febrile serositis and increased inflammatory markers [1]. The disease is associated with mutations in the FMF gene (MEFV), which encodes pyrin [2], an inflammasomal protein that, by being mutated, predisposes the inflammasome to produce interleukin 1, the cytokine underlying FMF and driving its attacks [3]. In the absence of diagnostic tests and due to the low sensitivity and specificity of genetic testing, the diagnosis of FMF is clinical and relies on typical general and site-specific features of the attacks [4–7].

Nevertheless, a proportion of FMF patients, displaying an incomplete and atypical clinical picture, are still diagnosed with FMF, giving rise to misdiagnosis of FMF instead of another disease with comparable manifestations. Furthermore, in addition to the typical attacks, FMF patients exhibit a wide spectrum of manifestations, such as chronic abdominal, joint and muscle pain, rash, diarrhea, peripheral neuropathy, nephropathy with proteinuria and hematuria. These manifestations either stem directly from FMF, arise from comorbidities commonly occurring in FMF (e.g., Behcet's disease, inflammatory bowel disease, glomerulonephritis, fibromyalgia, multiple sclerosis) or constitute adverse events of colchicine, the drug used to treat FMF [8–12]. Thus, patients suffering from FMF may manifest a myriad of signs, symptoms, and laboratory findings shared by other diseases, including Fabry’s disease (FD).

FD is a rare X-linked lysosomal storage disease caused by mutations in lysosomal *alpha-galactosidase A* (*GLA*), which metabolizes a sphingolipid called globotriaosylceramide (Gb3). The mutations may disrupt *GLA* function and lead to Gb3 accumulation in various tissues, mostly blood vessels, skin, kidneys, eyes, and central and peripheral nervous systems [13]. The resulting FD phenotype is highly nonspecific, comprising at least some of the following manifestations: abdominal pain, diarrhea, neuropathy, skin rash, renal failure with proteinuria, ischemic cardiovascular and central nervous system disease, fever, and more [14–17].

The diagnosis of FD usually requires a high index of suspicion in patients presenting with one or more of the nonspecific manifestations listed above and is based on chemical and genetic analyses, demonstrating *GLA* enzyme inactivity and *GLA* gene mutation. Because of a high rate of missed and delayed diagnoses, a practice has evolved to look for patients with FD in populations with high odds of finding latent FD, including in patients with undiagnosed kidney failure, young patients with cardiovascular disease, and patients with undiagnosed fever of unknown origin [18–21].

Because FMF and FD share common manifestations, FD may be misdiagnosed as FMF or may be missed as a concurrent disease in patients with FMF. Indeed, several studies have reported on spotting FD among patients with FMF [22, 23]. In the present study, we attempted to detect FD patients whose diagnosis was missed in a cohort of FMF patients. To improve the prospects of finding FD, we pursued FD in a subpopulation of FMF enriched for manifestations shared by the two diseases.

## Materials and methods

### Setting

The FMF clinic of the Chaim Sheba Medical Center at Tel Hashomer actively follows a population of approximately 4,000 patients. Patients are usually seen at 6- to 24-month intervals, unless urgent or more frequent visits are required. During the follow-up visits, patients’ data are collected in a structured computerized file constructed specifically for FMF patients.

### Study overview

The study was carried out between December 2016 and October 2020. During this period, 500 FMF patients who paid their regular follow-up visit to the Sheba FMF clinic were screened by one of the authors (AL) for appropriateness for the study. Those fulfilling all study inclusion and none of the exclusion criteria and who agreed to participate signed an informed consent form and were enrolled in the study. Each participant was examined and interviewed and provided a blood sample to test for FD. Given the definite diagnosis attained by our testing methodology (see next), we have not looked directly at physical features supporting FD and have not performed studies such as electrocardiogram, echocardiogram, slit lamp testing or urine tests during patient enrollment. The study was approved by the medical center’s institutional review board for human experimentation. All procedures were performed in accordance with the relevant guidelines and regulations.

### Data collection

A questionnaire devised for the study was completed for each patient. The questionnaire collected data on FMF-associated demographic, clinical, laboratory, genetic, and treatment parameters, as well as comorbidities and manifestations not directly related to FMF, but could potentially have stemmed from latent FD. Table 1 displays clinical features that were particularly sought to strengthen the likelihood of finding FD patients. These features were of two types: Category A—manifestations shared by FMF and FD, and Category B—features averting FMF from the typical presentation, thereby increasing the odds for a different diagnosis. Having at least one of these features was mandatory for inclusion in the study.

**Table 1:** Features increasing likelihood of spotting latent or misdiagnosed FD among FMF patients

Category	Group	Manifestation / feature	Definition of the manifestation / feature
A. Overlapping manifestations	A1. Typical manifestations of FMF	Proteinuria or kidney function impairment	Of unknown cause. Amyloid nephropathy was excluded by tissue biopsy, or atypical clinical course*
		Splenomegaly	Long axis is more than 13 cm by US imaging
		Recurrent episodes with fever alone	Unexplained fever episodes, not accompanied by abdominal, chest or joint pain, may be accompanied by constitutional manifestations**
	A2. Incomplete manifestations of FMF	Abdominal pain	Afebrile attacks, localized rather than diffused pain, longer than a week
		Joint involvement	Arthralgia, afebrile episodes, long duration (> week)
	A3. Manifestations of FMF comorbidities or colchicine adverse effects	Ischemic cardiovascular disease	MI, TIA, CVA in patients younger than 50 years
		Fibromyalgia-like pain	Particularly limb pain and generalized pain, without explanation, therefore labeled fibromyalgia
		Neuropathic pain	Including numbness paresthesia, burning pain without explanation
B. Features diverting the FMF from typical presentation	B1. Genetic testing	Genetic negative FMF	Absence of at least 5 most common *MEFV* mutations (M694V, V726A, E148Q, M680I, M694I)
	B2. Treatment experience	Colchicine failure	Lack of FMF response to at least 2 mg/day of colchicine, excluding intolerance or allergy to colchicine

US: ultrasound, CVA: cerebrovascular accident, FMF: familial Mediterranean fever, FD: Fabry’s disease, MEFV: Mediterranean fever gene, MI: myocardial infarction, TIA: transient ischemic attack

^*^Typical course of amyloid nephropathy—slowly growing proteinuria, usually reaching a nephrotic stage, followed by slow evolution of renal function loss

**Constitutional manifestations—chills, diffuse muscle pain, headache, tiredness, weakness, sweating.

### Inclusion criteria

A patient was enrolled in the study if he met all the following criteria:

1. Aged 18 years or older,
2. Diagnosed with FMF based on our widely used criteria for diagnosis of FMF [24],
3. Followed in the FMF clinic repeatedly for at least five years,
4. Experiencing or exhibiting one or more of the features listed in Table 1.

### Exclusion criteria

A patient could not be recruited to the study if he met any of the following criteria:

1. Unable to sign an informed consent,
2. Never underwent genetic testing for FMF,
3. Failed to attend clinic follow-up visits for 5 or more years,
4. Exhibiting none of the features outlined in Table 1.

### Testing for FD

A pinprick puncture of a finger and collection of a small blood drop onto a Guthrie card was performed in the FMF clinic. Testing of the dried blood spot sample for FD was performed at the Archimed laboratory (Vienna, Austria) and included analysis of enzyme activity (men), reference value > 1.2 micromole/liter/hour [25], and mutational analysis of the *GLA* gene, using sequencing of exons and their flanking regions (women), as described previously [26]. Being an X-linked disease, men with FD display a complete lack of enzyme activity, while women may present with either partial deficiency (usually) or complete deficiency if the second X-chromosome is inactivated or the second *GLA* allele is also mutated. It was planned that both genetic and enzymatic tests would be performed if one of the tests was positive or borderline. In patients with borderline enzyme activity, blood Gb3 metabolites (lyso-Gb3) were tested as well [27].

## Results

### Patient characteristics

**Fig. 1:**
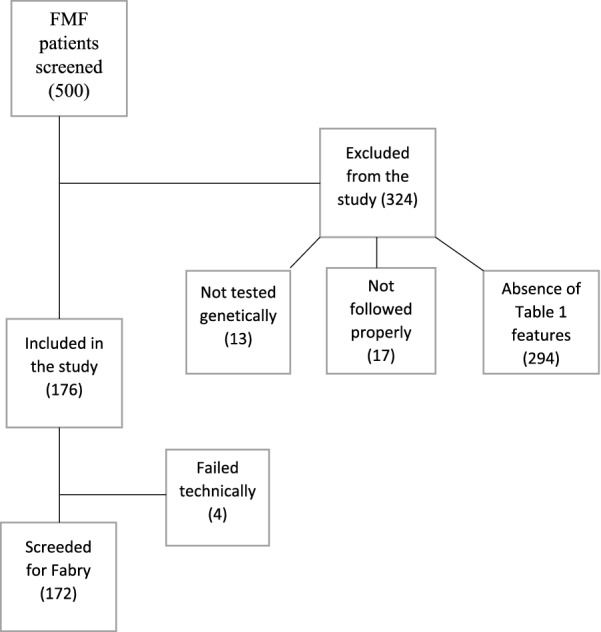
A flowchart sorting FMF patients screened for the study

Of the 500 FMF patients screened, 176 fulfilled the inclusion and none of the exclusion criteria. Figure 1 provides reasons for exclusion. Of the 176 patients enrolled, only 172 were tested for FD (four samples failed testing for technical reasons), 60% of whom were women. Table 2 shows the main FMF features of the studied patients. As might be expected, selection bias prompted by the inclusion criteria lessened the proportion of patients responding to colchicine to 30% and expanded the proportion of patients with various FMF-associated comorbidities to 48%, far beyond published rates in the general FMF population. Table 3 displays the actual distribution of the features specified in Table 1, favoring the capture of latent FD in the studied FMF patients. Most patients had more than one feature, bringing the number of features to a total of 358, of which 60 could be assigned to the group of manifestations of FMF shared by FD (A1), 75 to incomplete manifestations of FMF (A2), 83 to manifestations of comorbidities of FMF or of colchicine adverse effects (A3), 28 to negative genetic test for FMF (B1), and 112 to colchicine treatment failure (B2), which was the leading parameter prompting inclusion in the study.

**Table 2:** FMF features in a cohort of FMF patients evaluated for FD manifestations

Set	Feature	Finding
Demographic traits	Age at study entry—years, mean ± SD	47 ± 14
	Age at FMF onset—years, mean ± SD	13 ± 12
	Female sex—*n*, (%)	104, (60)
	Family history of FMF—*n*, (%)	128, (74)
Manifestations	Abdominal attacks—*n*, (%)	163, (95)
	Chest attacks—*n*, (%)	109, (63)
	Arthritis attacks—*n*, (%)	107, (62)
	Erysipeloid rash—*n*, (%)	17, (10)
	Acute scrotum—*n*, (%)	5, (3)
	Short-term muscle attacks—*n*, (%)	1, (1)
	Long-term muscle attacks—*n*, (%)	3, (2)
	Fever only attacks—*n*, (%)	12, (7)
	Leg pain—*n*, (%)	115, (67)
Associated comorbidities	Inflammatory bowel disease—*n*, (%)	3, (2)
	Ankylosing spondylitis—*n*, (%)	29, (17)
	Behçet’s disease—*n*, (%)	48, (28)
	Fibromyalgia—*n*, (%)	26, (15)
	Patients with any comorbidity—*n*, (%)	82, (48)
Treatment	Colchicine—*n*, (%)	164, (95)
	Mean colchicine dose—mg/day, mean ± SD	1.9 ± 0.84
	Colchicine dose ≥ 2 mg/day—*n*, (%)	103, (60)
	Response to colchicine- n, (%)	52, (30)
	Biologic drugs—*n*, (%)	15, (9)
Genetics	Two or more mutations—*n*, (%)	98, (57)
	One mutation—*n*, (%)	35, (20)
	No mutations in genetic testing—*n*, (%)	28, (16)
	M694V/M694V—*n*, (%)	65, (38)
	M694V/0—*n*, (%)	28, (16)

**Table 3:** Distribution in the study group of features favoring the detection of FD

Manifestation/feature	Group	Number (%)
Proteinuria*	A1	27 (16)
Renal failure	A1	5 (3)
Spleen enlargement	A1	16 (9)
Fever only	A1	12 (7)
Incomplete abdominal attacks	A2	16 (9)
Joint involvements which are not typical of FMF	A2	59 (34)
Fibromyalgia-like manifestations	A3	26 (15)
Neuropathy	A3	24 (14)
Cardiomyopathy / atherosclerotic heart disease	A3	14 (8)
Peripheral vascular disease	A3	5 (3)
CNS vascular disease	A3	14 (8)
Absence of mutations in genetic testing	B1	28 (16)
Lack of response to colchicine	B2	112 (65)

^*^In 3 patients, tissue biopsy was performed (kidney, gastric and liver), which excluded amyloid deposition. None was read as suspicious of FD, but routine light microscopy (H&E) may fail to detect FD, even retrospectively. All biopsied patients had features in addition to proteinuria that made them eligible for the study (neuropathy and fever in one, colchicine resistance and fever episodes in the second and stroke in young age and colchicine resistance in the third

### Main findings

Of 172 FMF patients with increased odds for latent FD, none had a positive test for FD.

## Discussion

In this study, we looked for latent FD in 172 FMF patients selected because of a higher risk of being misdiagnosed with FMF instead of FD or having FD in addition to FMF. However, despite exhibiting manifestations shared by FD, a profile different from typical FMF, or features representing both categories, none of the study patients tested positive for FD.

Our results do not concur with published reports on several FD cases that were recovered from cohorts of patients with FMF [22, 23, 28, 29]. However, most of these subjects do not fit into the paradigm that FMF may mask FD. In some, the diagnosis of FMF was clearly established mistakenly, as appears for instance in a patient who received a diagnosis of FMF based on nocturnal fever lasting several months, bilateral finger arthritis, and carriage of a nonpathogenic *MEFV* mutation [22]. Others either did not display manifestations of FD or had only a few mild symptoms, such as occasional paresthesia or mild “cardiac symptoms” [22], which, independent of FMF, will not elicit exploration for FD; therefore, the diagnosis of FD was not actually missed due to concordant FMF. In the remaining patients, the data provided are insufficient to determine whether they should have received a diagnosis of FMF or FD to begin with [22, 23].

There have been at least two published cases in which FD was identified in patients who fulfilled the criteria for FMF [26, 27]. Per the data available, the diagnosis of FMF rested on both recurrent short episodes of febrile abdominal attacks and FMF genetic testing. Later, FD was diagnosed in one case based on kidney biopsy performed for proteinuria and then on *GLA* activity, which was nil, and in the other case, on clinical, chemistry and genetic analysis for FD. Thus, concealing of FD by FMF does occur and contradicts our findings. Moreover, carriage of FMF mutations does not relieve the diagnostic confusion between FD and FMF, as implied by four of five FMF-FD cases who tested positive for FMF mutations [22, 23, 29]. Thus, despite the flaws formerly presented, altogether, published data support viewing FMF as a risk factor leading to miss a diagnosis of FD.

Experience gained in other high-risk populations, suggests that exploratory trials screening patients for FD is usually beneficial. For instance, Fancellu et al. reported a 41-year-old male, identified among 178 young patients with neurovascular disorders. The patient was found to carry the R227Q *GLA* mutation although presented only with white matter lesions [30]. Other beneficial probing of at risk populations is frequently reported in patients with chronic kidney disease, stroke, cardiac hypertrophy, and other population [18–21, 31, 32]. However, negative screenings may also occur [33, 34], making our results not an exception.

Three possible explanations might be applied to our nonsuccess to detect even one case:

1. Sample size—The prevalence of FD in the general population as determined by newborn screening may reach 1:7000 infants with pathogenic mutations, mostly causing late onset disease [35–37]. Therefore, to find even one case of FD among the 172 patients studied, FMF must enrich the odds by a factor of 40, which is clearly much more than expected from FMF. More specifically, if FMF doubles or even triples the risk of hiding FD disease, one needs approximately 2500 high-risk FMF patients (as defined in this study) to find 1 case of FD. Obviously, such a large cohort was not available to us even in the setting of a referral center.
2. Setting—In an FMF-dedicated clinic, misdiagnosis of FD as FMF is highly unlikely, as for experts, the diagnosis of FMF is based on deep recognition of the disease and its fine features.
3. Comorbidities of FMF—Although FD might be masked more readily in FMF patients affected by additional inflammatory disorders, most FMF patients with inflammatory comorbidity typically suffer from characteristic FMF and therefore are less likely to be misdiagnosed. Altogether, published data combined with our findings favor screening FMF for FD. This, however, should be done in less typical patients, who might be available in rheumatology clinics not solely dedicated to FMF

## Conclusions

In conclusion, in a study of 172 FMF patients selected by virtue of an increased risk of including FD patients misdiagnosed as FMF or with both FMF and overlooked FD, we failed to detect even a single patient with FD. This may result from the strategy of the study, though it more likely reflects the high standard of FMF diagnosis found in an FMF-dedicated clinic. Based on published data and the interpretation of our negative findings, FMF remains a risk factor for latent FD. However, a search for FD should be focused on FMF patients managed in clinics not uniquely specialized in FMF, or on new referrals presented to an FMF-dedicated clinic with manifestations overlapping with those of FD (e.g., kidney disease, leg pain, abdominal pain, etc.), who fail a diagnosis of FMF.

## Data Availability

The datasets used and/or analyzed during the current study are available from the corresponding author on reasonable request.

## Abbreviations

CVA: Cerebrovascular accident
FD: Fabry’s disease
FMF: Familial Mediterranean fever
Gb3: Globotriaosylceramide
*GLA*: *Alpha-galactosidase A*
*MEFV*: Mediterranean fever gene
MI: Myocardial infarction
TIA: Transient ischemic attack
US: Ultrasound
